# Comparison of microfluidic platforms for the enrichment of circulating tumor cells in breast cancer patients

**DOI:** 10.1007/s10549-022-06717-x

**Published:** 2022-09-08

**Authors:** Constantin Sajdik, Eva Schuster, Barbara Holzer, Michael Krainer, Christine Deutschmann, Stefan Peter, Maximilian Marhold, Robert Zeillinger, Eva Obermayr

**Affiliations:** 1 Department of Obstetrics and Gynecology, Comprehensive Cancer Center, Medical University of Vienna, Waehringer Guertel 18-20, 1090 Vienna, Austria; 2 Landesklinikum Hochegg, Pulmologische Abteilung, Hocheggerstraße 88, 2840 Grimmenstein, Austria; 3 Department of Medicine I, Clinical Division of Oncology, Comprehensive Cancer Center, Medical University of Vienna, Waehringer Guertel 18-20, 1090 Vienna, Austria; 4 ANGLE Europe Limited, 10 Nugent Road, Surrey Research Park, Guildford, GU2 7AF Surrey UK

**Keywords:** Liquid biopsy, Gene expression analysis, Microfluidic enrichment, Density gradient centrifugation, Early breast cancer

## Abstract

**Purpose:**

Circulating tumor cells (CTCs) hold promise to be a non-invasive measurable biomarker in all cancer stages. Because the analysis of CTCs is still a technical challenge, we compared different types of microfluidic enrichment protocols to isolate these rare cells from the blood.

**Methods:**

Blood samples from patients with early and metastatic breast cancer (BC) were processed using the microfluidic Parsortix® technology employing (i) a single-step cell separation using the standard GEN3D6.5 microfluidic cassette, (ii) a two-step separation with an upfront pre-enrichment, and (iii) a two-step separation with a different type of cassette. In the enriched cells, the gene expression levels of CTC-related transcripts were assessed using quantitative real-time PCR (qPCR) by Taqman® and Lightcycler (LC) technology.

**Results:**

23/60 (38.3%) BC samples were assigned as positive due to the presence of at least one gene marker beyond the threshold level. The prevalence of epithelial markers was significantly higher in metastatic compared to early BC (*EpCAM*: 31.3% vs. 7.3%; *CK19*: 21.1% vs. 2.4%). A high level of concordance was observed between *CK19* assessed by Taqman® and LC technology, and for detection of the BC-specific gene *SCGB2A2*. An upfront pre-enrichment resulted in lower leukocyte contamination, at the cost of fewer tumor cells captured.

**Conclusion:**

The Parsortix® system offers both reasonable recovery of tumor cells and depletion of contaminating leukocytes when the single-step separation using the GEN3D6.5 cassette is employed. Careful selection of suitable markers and cut-off thresholds is an essential point for the subsequent molecular analysis of the enriched cells.

## Introduction

The analysis of circulating tumor cells (CTCs) is usually a two-step procedure, consisting of enrichment and detection, both being critical for the specificity and sensitivity of the overall approach. Regardless of whether CTCs are detected and further characterized by their intracellular proteins, gene expression pattern or genetic make-up, the background of benign cells may interfere with the analysis and thus needs to be substantially reduced.

The number of residual white blood cells (WBC) is of particular relevance when CTCs are detected at the transcriptional level, because a number of genes may also be expressed in leukocytes, although to a very small extent. In this regard, the Parsortix® technology (Angle plc., UK), which relies on the microfluidic separation of cells due to their larger size and/or limited deformability as compared to blood cells, proved to reduce the cellular background to a sufficient extent [4]. Only recently, the Parsortix® technology was cleared by the US Food and Drug Administration for the diagnosis of CTCs in metastatic breast cancer. Important features are preserved viability of the enriched cells, high depletion of WBC, independence from antigens on the cell surface, and automated and fast processing of large blood volumes up to 50 ml. The microfluidic separation of cells of interest takes place in a microscope slide-sized disposable cassette which is mounted in the Parsortix® PR1 device that generates controlled fluid flow during separation. The sample (e.g., whole blood) passes through a fluidic path over a stepped separator, and cell capture is defined by the geometry (height) of the final step. Thus this so-called “critical gap size” is a relevant parameter of the specific cassette types, impacting cell recovery and residual WBC count, and finally duration of the entire separation process.

Recently, it was shown that Parsortix® enabled specific detection of CTC-related transcripts with a rate of nearly 100%; however, improvements of tumor cell recovery rate should be sought [10]. Meanwhile, the manufacturer modified the microfluidic cassette by reducing the critical gap size from 10 µm (GEN3D10 separation cassette) to 6.5 µm (GEN3D6.5 separation cassette), claiming to enable detection of smaller cells of interest, and consequently increase overall recovery of target cells. Thus, due to its’ smaller gap size, the GEN3D6.5 cassette could allow for isolation of smaller CTCs; however, with the potential caveat of higher numbers of residual WBCs.

In the present study, the transcript levels of a 6-gene panel (*CCNE2*, *PPIC*, *MAL2*, *EMP2*, *HJURP*, and *SCL6A8*) previously published as candidate CTC markers [11], and of epithelial cell lineage-specific (*EpCAM*, *CK19*) and breast cancer-specific (*SCGB2A2*) markers were assessed. Blood samples from patients with early and metastatic breast cancer (BC) after single-step enrichment using the Parsortix® GEN3D6.5 separation cassette were processed. Furthermore, the performance of the single-step separation with a previously developed two-step protocol, employing the GEN3D10 cassette with a larger critical gap of 10 µm and an upfront density gradient (DG) pre-enrichment at a lower flow rate was assessed [10]. Finally, the question was asked whether another two-step enrichment, namely combining DG and the GEN3D6.5 microfluidic cassette, would sufficiently reduce the number of WBCs without relative loss of cells within the target cell population.

## Materials and methods

### Patients

Blood samples were taken from treatment-naïve BC patients at the Department of Obstetrics and Gynecology and at the Division of Oncology, Department of Medicine I (both at Vienna General Hospital, Austria). Patient samples were defined as early (stage I and II) and metastatic (stage IV) BC based on the Tumor Node Metastasis (TNM) classification. Control blood samples were collected from female healthy donors (HD) without known history of cancer.

### Processing of the blood samples

**Fig. 1:**
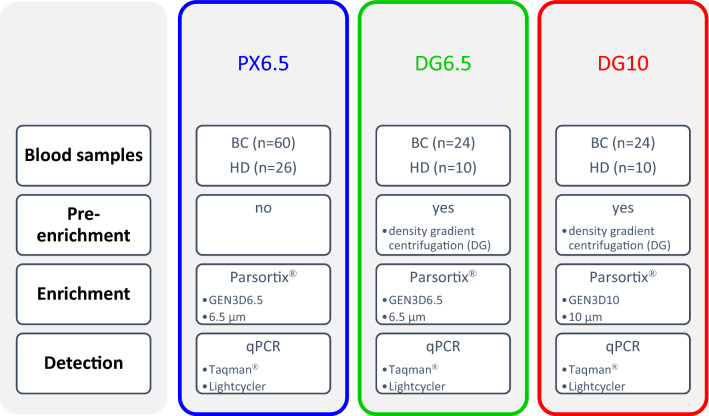
Flow diagram of the protocols applied for the enrichment of circulating tumor cells (CTCs) and the detection of CTC-related gene transcripts. CTCs were enriched using the microfluidic Parsortix® enrichment alone and in combination with an upstream density gradient centrifugation. CTC-related gene transcripts were detected using Taqman® and Lightcycler technology (LC)

Thirty ml of blood was collected in Vacuette K3EDTA tubes (Greiner Bio-One) and processed on the same day in accordance with a recently published protocol [10]. In short, the blood was divided into two equal parts to enable comparison of single-step and two-step enrichment of CTCs. Using the single-step protocol (PX6.5), Parsortix® was the sole enrichment step employing the GEN3D6.5 microfluidic cassette with the critical gap size of 6.5 µm. With the two-step protocols, the blood samples were first enriched by DG centrifugation using 15 ml Percoll (GE Healthcare; *d* = 1.065 g/ml, 305 mOsm/kg), and then, the cell suspensions were further processed with GEN3D10 cassettes applying 23 mbar pressure (DG10), or GEN3D6.5 cassettes applying 99 mbar pressure (DG6.5). An overview on the respective protocols is given in Fig. 1. After the microfluidic separation, the captured cells were harvested and immediately lysed by adding 350 µl RLT lysis buffer (Qiagen).

### Spiking experiments

The breast cancer cell line SKBR-3 and the ovarian cancer cell line CaOV-3 were grown in RPMI-1640 supplemented with 10% fetal bovine serum and 1% penicillin–streptomycin–gentamicin (Invitrogen) in a humidified atmosphere at 37 °C and 5% CO2. At about 70% confluence, the cells were trypsinized and stained with CellTrace Violet (Invitrogen). One-hundred stained cells were manually picked under a microscope and added to a 15 ml HD blood sample, which was then processed using the respective protocol as described above. In order to assess the capture rate of the microfluidic enrichment, the fluorescently labeled tumor cells trapped within the microfluidic cassette were counted by two independent observers. The amount of residual WBC in the harvest was assessed using a Neubauer counting chamber. Then, as described above, the harvested cells were lysed for subsequent RNA extraction.

The efficiency of the single-step microfluidic protocol PX6.5 to capture SKBR-3 cancer cells was assessed in 12 replicate sample and six Parsortix® PR1 devices (two samples per device). The performance of PX6.5 and two-step enrichment protocols DG6.5 and DG10 was compared by processing each five replicate HD blood samples spiked with the CaOV-3 cell line.

### RNA extraction and reverse transcription

Total RNA was extracted from the cell lysates using the RNeasy Micro Kit (Qiagen) without DNase treatment. The total amount of RNA was converted into cDNA using the SuperScript VILO Mastermix (Invitrogen), half of which was further analyzed using gene-specific pre-amplification and Taqman®-based qPCR. The other half was analyzed using *CK19*-specific primers and hybridization FRET (Fluorescent Resonance Energy Transfer) probes.

### Gene expression analysis with qPCR

Following gene-specific pre-amplification, qPCR was performed in duplicates using TaqMan® Universal Mastermix II and exon spanning TaqMan® assays (*EpCAM*, *CCNE2*, *PPIC*, *MAL2*, *EMP2*, *HJURP*, *SCGB2A2*, and *SCL6A8*; Life Technologies). *CDKN1B* was chosen as reference gene to control for sample quality and quantity. qPCR was performed on the ViiA7 Real-Time PCR System with standard thermal cycling parameters. *CK19*-specific qPCR was done using published primer sequences [16] and with a FAM-labeled hydrolysis probe (5'-TgTCCTgCAgATCgACAACgCCC-3´). Raw data were analyzed using ViiA7 Software (v1.1) with automatic threshold setting and baseline correction. In addition to the Taqman®-based qPCR, *CK19* transcripts without prior pre-amplification were assessed using primers and hybridization FRET probes according to Stathopoulou et al. [16] on a Lightcycler 480 instrument (Roche).

### Calculation of cut-off threshold values

If the amplification curve did not reach the threshold line in both duplicate reactions, the sample was regarded as negative for that respective transcript. Similarly, mean Ct values ≥ 35.0 were set to “undetermined” and regarded as absent gene expression. For every marker with undetectable gene expression in HD samples, any mean Ct-value in patient samples < 35.0 was assigned as positive.

In contrast, for every marker with gene expression detected in HD samples (i.e. Ct-value < 35), a cut-off value was calculated by adding the x-fold standard deviation (SD) to the mean of the positive HD samples, so that the overall false positive rate in the HD group became lower than 10% [2]. This calculation was done for each gene marker separately. A patient sample was then assigned positive for the respective gene marker if the Ct-value was below the calculated threshold and negative if the Ct-value was beyond the threshold.

### Statistics

Residual WBCs, recovery of spiked tumor cells, and duration of microfluidic enrichment by the respective protocols are presented as mean and standard deviation (SD) from replicate experiments. An ordinary one-way ANOVA was performed to evaluate differences between respective enrichment protocols. The association between captured and harvested tumor cells was assessed using Pearson correlation. The Fisher Exact test was performed to examine the relation between marker positivity and stage of disease at time of blood draw (early vs. metastatic BC). Differences in positivity rates between the respective enrichment protocols were assessed using McNemar test. To evaluate the level of concordance between the single-step (PX6.5) and two-step enrichment (DG6.5 and DG10) the Kappa test adjusted for low prevalence and bias was used [14], and the positive negative percent agreement (PPA and NPA) was calculated. Statistical analyses was performed using GraphPad Prism (vs. 9.3.1). The level of significance was set at *p* < 0.05.

## Results

### Performance of microfluidic enrichment as a single-step process and in combination with density gradient centrifugation

With the single-step approach PX6.5 the overall mean capture rate of SKBR-3 was 59.9% (*n* = 12, SD ± 7.06; 95% CI 55.4–64.4), and all six devices performed similarly (two-way ANOVA, *p* = 0.054). A strong correlation was observed between the number of captured and harvested cells (Pearson’s *r* = 0.8115; *p* = 0.001).

**Fig. 2:**
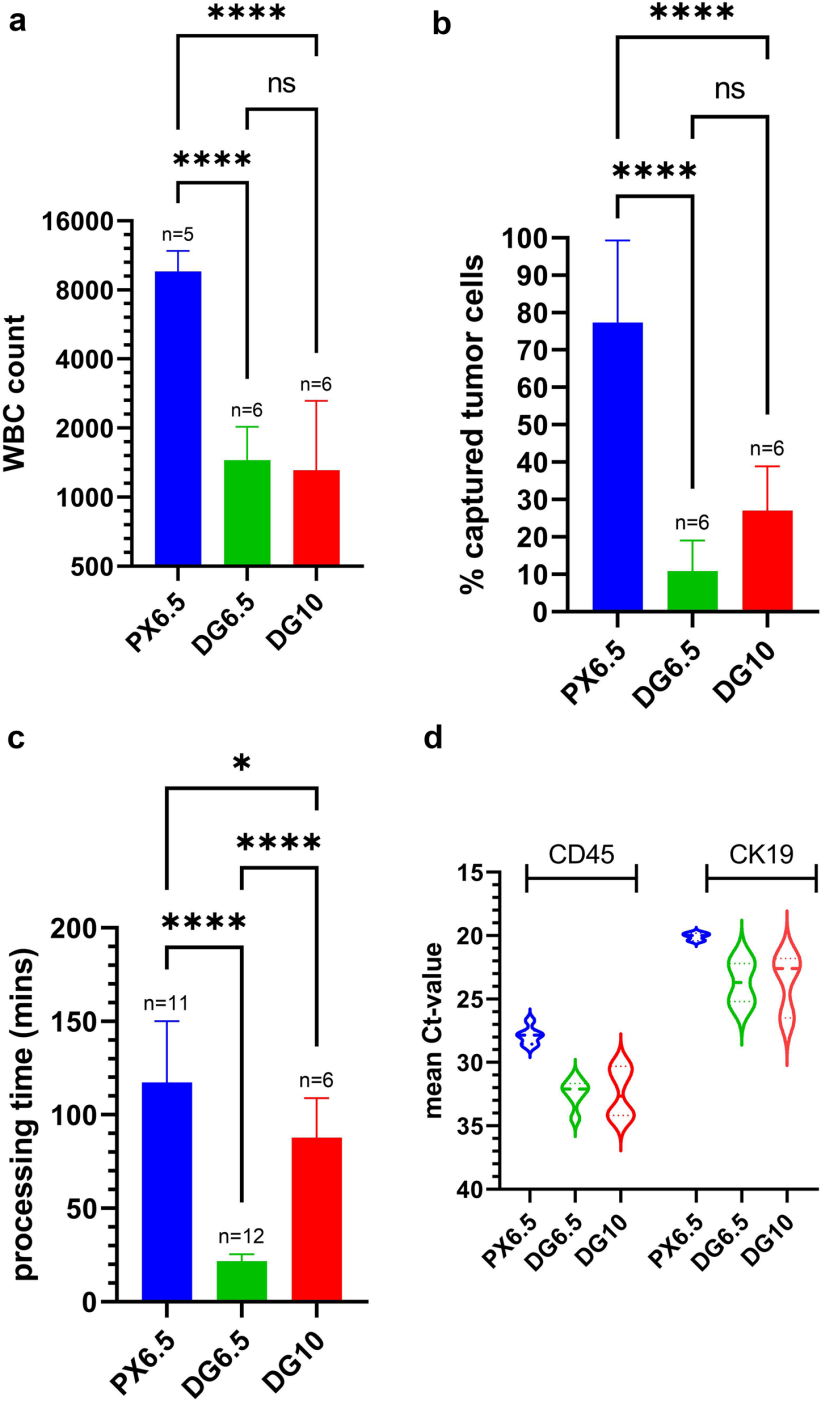
Characteristics of single-step and two-step enrichment protocols. **a** Number of residual WBCs after enrichment; **b** CaOV-3 tumor cell recovery; **c** duration of the respective protocols. Bars depicting mean and the error bar standard deviation of replicate spiking experiments, with a one-way ANOVA assessing the difference of the respective protocols. **d** Violin plot showing Ct-values of leukocyte-specific *CD45* and of epithelial cell-specific *CK19* of harvested cells enriched by respective protocols. Statistical comparisons are expressed with asterisks (*p ≤ 0.05, ****p ≤ 0.0001). ns non-significant

The standard procedure PX6.5 starting from whole blood was characterized by largest number of WBCs in the harvest (mean 9644 WBCs, SD ± 2191; Fig. 2a) and the highest capture rate (mean 77.4 CaOV-3 cells, SD ± 21.9; Fig. 2b). However, PX6.5 took significantly longer than the separation of pre-enriched samples, which in turn took significantly longer with DG10 than with DG6.5 (Fig. 2c). The differences in WBC content and tumor cell recovery between the respective protocols was further reflected by gene expression levels of the leukocyte-specific gene *CD45* and the epithelial cell marker gene *CK19* (Fig. 2d).

### CTC-related markers following the single-step microfluidic approach PX6.5

To answer the question, whether the number of residual leukocytes after the single-step PX6.5 protocol was still suitable for subsequent qPCR, and whether the discrimination of blood samples potentially harboring CTCs is feasible, the gene expression levels of selected markers in blood samples from 26 female HD and 60 BC patients were assessed. In none of the control samples, *CK19*, *MAL2* and *SCGB2A2* transcripts were observed; thus, any BC samples with a Ct-value < 35 was assigned as positive for the respective marker. *EpCAM* and *PPIC* were detected in six (23.1%) samples each, *EMP2* in ten (38.5%), and *HJURP*, SLC6A8, and *CCNE2* in all HD samples. For each of these genes, a cut-off threshold value was calculated in order to identify BC blood samples with transcript levels beyond background due to contaminating leukocytes.

**Fig. 3:**
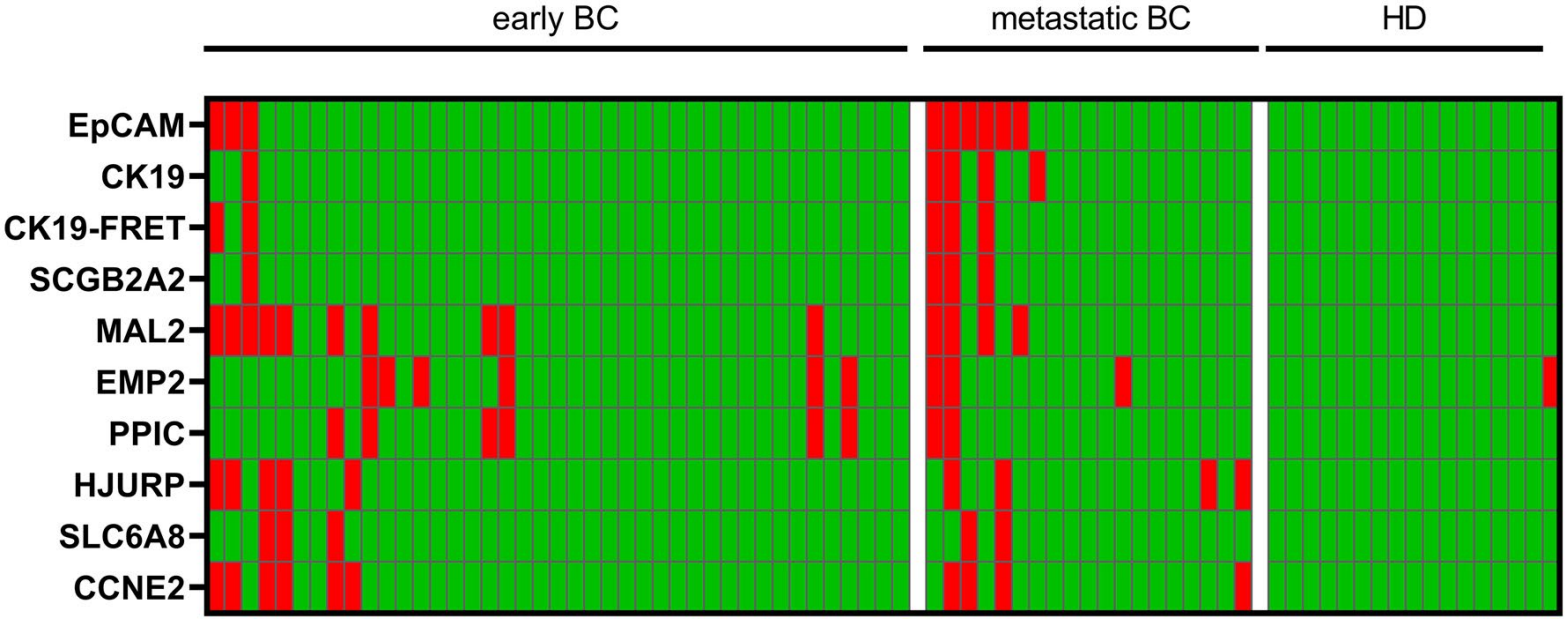
Heat map showing the prevalence of transcripts. Red squares indicate gene expression beyond calculated threshold level per tested sample in patients with early and metastatic BC, and in HD

In BC patient samples, transcript levels of at least one gene beyond the calculated threshold value were observed in 23/60 (38.3%) of clinical specimen (Table 1 and Fig. 3). Pre-amplified gene expression levels of *EpCAM* and *CK19* exhibited significantly higher prevalence in metastatic BC when compared with early BC (*EpCAM*: 31.3% vs. 7.3%; *CK19*: 21.1% vs. 2.4%). Among healthy controls, 1/26 samples had a gene expression level (of *EMP2*) beyond the selected cut-off (Fig. 3), indicating a high specificity.

**Table 1:** Prevalence of gene expression levels beyond the calculated threshold in samples from BC patients and healthy donors

	All BC (*n* = 60)	early BC (*n* = 41)	metastatic BC (*n* = 19)	*p*	HD (*n* = 26)
Overall	23 (38.3%)	14 (34.1%)	9 (47.4%)	0.397	1 (3.8%)
*EpCAM*	9 (15.0%)	3 (7.3%)	6 (31.6%)	0.023	0
*CK19*	5 (8.3%)	1 (2.4%)	4 (21.1%)	0.031	0
*CK19*-FRET	5 (8.3%)	2 (4.8%)	3 (15.8%)	0.314	0
*SCGB2A2*	4 (6.7%)	1 (2.4%)	3 (15.8%)	0.089	0
*MAL2*	14 (23.3%)	10 (24.4%)	4 (21.1%)	1	0
*EMP2*	9 (15.0%)	6 (14.6%)	3 (15.8%)	1	1 (3.8%)
*PPIC*	8 (13.3%)	6 (14.6%)	2 (10.5%)	1	0
*HJURP*	9 (15.0%)	5 (12.2%)	4 (21.1%)	0.445	0
*SCL6A8*	5 (8.3%)	3 (7.3%)	2 (10.5%)	0.648	0
*CCNE2*	10 (16.7%)	6 (14.6%)	4 (21.1%)	0.711	0

Absolute and relative numbers of positive findings are shown for the total study population of 60 patients, and stratified by stage of disease. The Fisher Exact Test was performed to examine the relation between marker positivity and stage of disease at time of blood draw (early vs. metastatic)

Using a published protocol employing highly specific *CK19* primers and FRET probes without prior pre-amplification, *CK19* transcripts were detected in none of the 26 HD samples, but in 5/60 BC samples (4/19 (21.1%) metastatic BC, 2/41 (4.9%) early BC). A substantial level of concordance of Taqman® and LC technologies was observed (Cohen’s *κ* = 0.74; 95% CI 0.34–1.16), as well as of *CK19*- and *SCGB2A2*-positivity (Cohen’s *κ* = 0.86; 95% CI 0.46–1.27).

### CTC-related markers following the combined enrichment (DG6.5) using density gradient centrifugation and the GEN3D6.5 microfluidic cassette

To find out whether further depletion of WBCs would increase the specificity of qPCR-based detection of the selected markers compared to single-step enrichment, the single-step microfluidic enrichment using a GEN3D6.5 cassette was compared with two-step enrichment using preceding DG centrifugation in paired blood samples from 24 BC patients (early BC *n* = 8, metastatic BC *n* = 16).

**Fig. 4:**
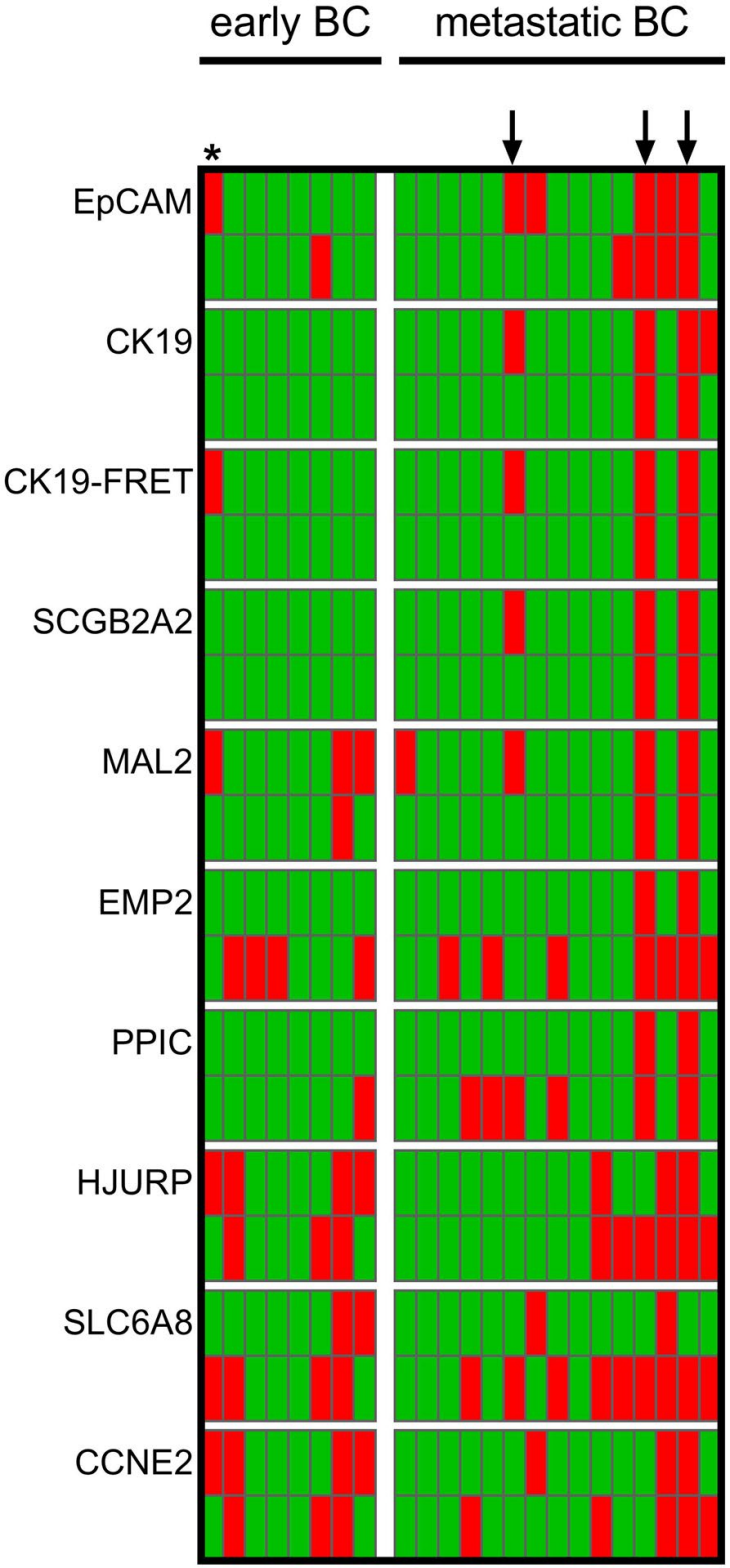
Prevalence of transcripts in BC patients with early (left panel) and metastatic (right panel) disease. Gene expression beyond the cut-off is indicated in red, below the cut-off in green. For each marker, the prevalence obtained by the single-step enrichment is shown in the upper row (PX6.5), whereas the bottom row the prevalence after an additional pre-enrichment (DG6.5) is shown. Arrows and the asterisk point to patients with a high number of positive gene markers

Overall, 10 of 12 PX6.5-positive samples remained positive after combined enrichment (PPA 83.3%). *EpCAM*, *CK19*, and *SCGB2A2* are characterized by high negative and only a moderate positive agreement (NPA 88.2–100.0%; PPA 42.9–66.7%) of single-step and two-step enrichment, suggesting high specificity of the markers used and better enrichment of CTCs using the PX6.5 standard protocol (Table 2). In three samples taken from metastatic BC patients (Fig. 4, highlighted by arrows), the presence of all epithelial markers may indicate a substantial number of CTCs, which may also be the reason for high concordance between the two enrichment methods in these cases. The co-emergence of *EpCAM*, *CK19*, and *MAL2* in just a single patient with early BC (Fig. 4, highlighted by an asterisk) may indicate a lower number of CTCs that can only be detected after single-step enrichment. *EMP2*-, *PPIC*-, *HJURP-*, and *SCL6A8*-positive samples are more likely to be observed after combined enrichment, thus suggesting that a higher number of residual WBCs after single-step enrichment affects cut-off threshold values and detection of CTCs.

**Table 2:** Prevalence of gene expression levels beyond the calculated threshold in paired BC blood samples (n = 24) enriched by single-step (PX6.5) and two-step approaches using upfront DG centrifugation (DG6.5)

	PX6.5	DG6.5			Concordance		
		Pos	Neg	*p*	PPA	NPA	*κ*
Overall	Pos Neg	10 8	2 4	0.114	83.3	33.3	0.17
*EpCAM*	Pos Neg	3 2	4 15	0.683	42.9	88.2	0.50
*CK19*	Pos Neg	2 0	2 20	0.480	50.0	100.0	0.83
*CK19*-FRET	Pos Neg	2 0	2 20	0.480	50.0	100.0	0.83
*SCGB2A2*	Pos Neg	2 0	1 21	1	66.7	100.0	0.92
*MAL2*	Pos Neg	3 0	4 17	0.134	42.9	100.0	0.67
*EMP2*	Pos Neg	2 9	0 13	0.008	100.0	59.1	0.25
*PPIC*	Pos Neg	2 5	0 17	0.074	100.0	77.3	0.58
*HJURP*	Pos Neg	5 4	2 13	0.683	71.4	76.5	0.50
*SCL6A8*	Pos Neg	2 11	2 9	0.027	50.0	45.0	-0.08
*CCNE2*	Pos Neg	4 4	3 13	1	57.1	76.5	0.42

Differences in positivity were assessed using the McNemar test. Additionally, the percent positive and negative agreement (PPA and NPA) of DG6.5 are shown (with the PX6.5 as reference), as well as the prevalence-adjusted bias-adjusted kappa

### Comparison of the previously employed combined protocol (DG10) and single-step microfluidic approach (PX6.5)

**Fig. 5:**
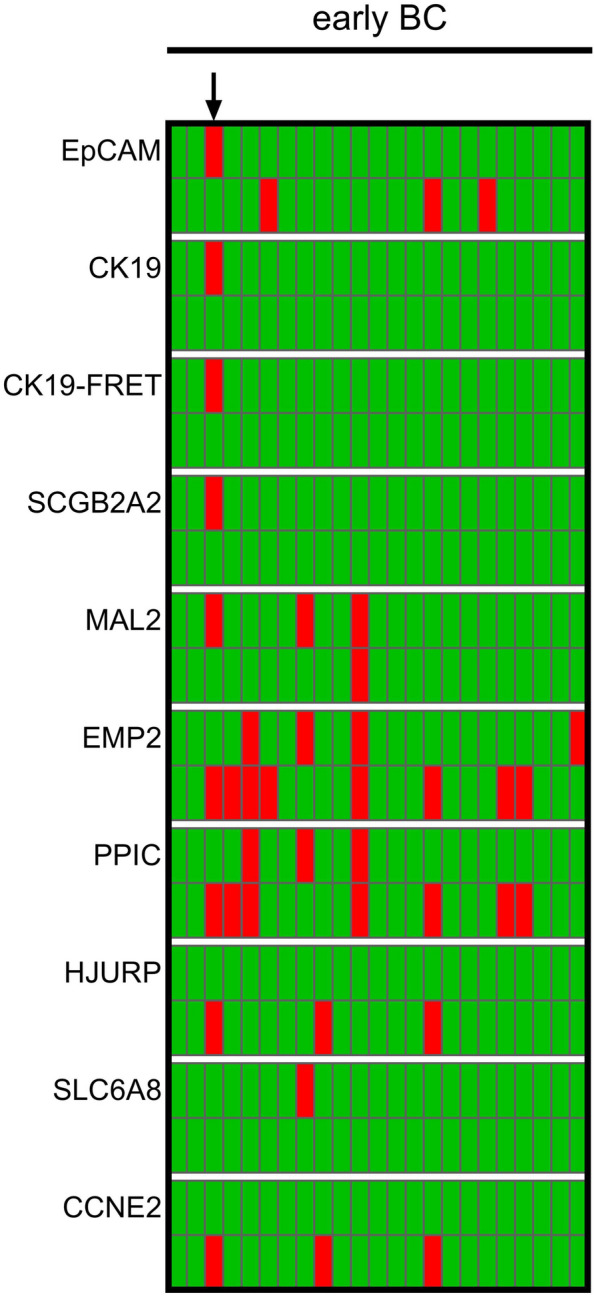
Prevalence of in BC patients with early disease. For each marker, the prevalence obtained by the standard enrichment using the GEN3D6.5 microfluidic cassette (PX6.5) is shown in the upper row, whereas the bottom row the prevalence after the previously established protocol using a 10 µm cassette and an additional pre-enrichment (DG10) is shown. Transcript levels beyond threshold cut-off level are marked in red. The arrow points to a patient with a high number of positive gene markers

In a subset of 24 paired blood samples from early BC patients, the standard single-step PX6.5 enrichment was compared to the previously used DG10 two-step protocol [10]. However, the small number of positive samples made it difficult to compare the approaches used (Table 3). None of the three *EpCAM*-positive samples enriched by DG10 was positive for *SCGB2A2* or *CK19*, which may indicate a low number of CTCs in these samples (Fig. 5). In contrast, employing the PX6.5 standard protocol, a single sample (Fig. 5, highlighted by an arrow) was positive for all epithelial markers and *MAL2*. The presence of epithelial CTCs in that specific sample was further indicated by *CK19* transcripts detected by highly specific *CK19* primer/FRET probes. These results endorse the higher sensitivity of the PX6.5 protocol. Overall, a poor concordance between the approaches was observed, with just 4/24 samples assigned as positive in both approaches due to transcript levels of at least one gene marker beyond the threshold (prevalence-adjusted bias-adjusted *κ* = 0.33).

**Table 3:** Prevalence of gene expression levels beyond the calculated threshold in paired BC blood samples (*n* = 24) enriched by standard (PX6.5) and combined approach using an upfront DG centrifugation and a different type of microfluidic cassette (DG10)

	PX6.5	DG10			Concordance		
		Pos	Neg	*p*	PPA	NPA	*κ*
Overall	Pos Neg	4 7	1 12	0.077	36.4	92.3	0.33
*EpCAM*	Pos Neg	0 3	1 20	0.617	0	95.0	0.67
*CK19*	Pos Neg	0 0	1 23	1	n.a	95.8	0.92
*CK19*-FRET	Pos Neg	0 0	1 23	1	n.a	95.8	0.92
*SCGB2A2*	Pos Neg	0 0	1 23	1	n.a	95.8	0.92
*MAL2*	Pos Neg	1 0	2 21	0.480	100.0	91.3	0.83
*EMP2*	Pos Neg	3 6	2 14	0.289	33.3	87.5	0.36
*PPIC*	Pos Neg	2 5	1 16	0.221	28.6	94.1	0.50
*HJURP*	Pos Neg	0 3	0 21	0.248	0	100.0	0.75
*SCL6A8*	Pos Neg	0 0	1 23	1	0	95.8	0.92
*CCNE2*	Pos Neg	0 3	0 21	0.248	0	100.0	0.75

Differences in positivity were assessed using the McNemar test. Additionally, the percent positive and negative agreement (PPA and NPA) of the standard approach are given (with DG10 as reference), as well as the prevalence-adjusted bias-adjusted kappa

## Discussion

Previously, it was shown that microfluidic enrichment of blood samples using the Parsortix® technology is compatible with gene expression analysis of enriched cells due to the highly efficient depletion of WBCs by the GEN3D10 cell separation cassette [10]. The question addressed by this study is whether reducing the critical gap size of the Parsortix® cell separation cassette from 10 to 6.5 µm would have an impact on subsequent molecular analyses using qPCR. In addition to established CTC markers (*EpCAM*, *CK19*, and *SCGB2A2*), a 6-gene panel potentially related to CTCs (*CCNE2*, *PPIC*, *MAL2*, *EMP2*, *HJURP*, and *SCL6A8*) was chosen. These genes were differentially expressed in 40 human cancer cell lines as compared to healthy donor blood samples and had been evaluated as candidate markers for the identification of CTCs in density gradient enriched patient samples [11].

The main finding of the present study is that the single-step microfluidic enrichment of whole blood using a critical gap of 6.5 µm represents a balanced compromise between tolerable loss of target cells and reasonable amount of residual WBCs. While the detection of highly specific transcripts such as *CK19* and *SCGB2A2* remains unaffected by the amount of WBC in the given setting, other CTC-related markers, such as *MAL2*, *EMP2*, and *PPIC* require a thorough evaluation of appropriate threshold values, which may then justify the association of elevated transcript levels and the presence of CTCs. The association of the gene markers comprised in the 6-gene panel with tumor cells was demonstrated in tissue specimen of cancer patients in an earlier study; however, the large number of residual WBC after density gradient enrichment compromised the association with CTCs in patient blood samples [11].

In addition to gene expression analysis using Taqman®-based qPCR following target-specific pre-amplification, a highly specific assay for the detection of *CK19* transcripts in CTCs using the hybridization probe-based Lightcycler technology was employed [16]. In line with Strati et al. and using their set of primers and probes in metastatic BC [17], 21.1% CK-positive cases in the same setting were observed. Moreover, the results from the Lightcycler qPCR reasonably agree with the Taqman® hydrolysis probe-based common qPCR in detecting *CK19* transcripts (Cohen’s *κ* = 0.74), alleviating concerns regarding a potential bias and lower specificity introduced by the target-specific pre-amplification step preceding Taqman® qPCR.

Furthermore, the presence of *CK19* was accompanied by *SCGB2A2* transcripts in 4/5 cases (Cohen’s *κ* = 0.86), providing further evidence for the validity of the findings obtained by a target-specific pre-amplification prior to qPCR. *SCGB2A2* is considered to be a mammary-specific epithelial marker that was found to be a valuable diagnostic tool to identify BC micrometastases in lymph nodes [20]. Bredemeier et al. showed that about one third of *CK19*-positive CTCs were characterized by *SCGB2A2* co-expression [1], while an inverse ratio was found by Van der Auwera et al., who further demonstrated the superiority of a multi-marker qPCR assay over *EpCAM*-based AdnaTest or CellSearch Assay [19]. The higher sensitivity of a multi-marker approach postulated in the latter study may be of relevance especially in early stages of cancer. Here, not only the overall sensitivity may be higher than with a single marker alone but also a score calculated based on the number of markers beyond the cut-off threshold may permit conclusions on CTC numbers or may be associated with outcome [5, 9, 15].

While numerous studies have described the clinical relevance of CTCs in metastatic BC, their role has yet to be established in early BC. In patients with surgically resectable disease, CTC counts and incidence are even lower than in patients with metastasized tumors, usually less than 1 CTC per 10 ml of blood [18]. These low numbers represent a major technical difficulty in CTC detection, and may make comparative studies more difficult or even impossible due to Poisson distribution of rare events. Similar to Politaki et al. [12], a higher concordance of different technologies was observed in the subset of metastatic BC samples, which had been processed using the same type of microfluidic cassette (GEN3D6.5) in both single-step and two-step enrichment. In contrast, none of the paired early BC samples processed by different types of cassettes (single-step GEN3D6.5 or two-step GEN3D10) was assigned positive by both methods: Here, only the single-step enrichment using GEN3D6.5 was able to yield a qPCR-positive result indicating higher transcript levels of epithelial markers in a single sample (arrow in Fig. 4, Table 3). However, these observations should be interpreted with caution because of small sample size and heterogeneity of blood samples used to compare standard microfluidic enrichment with modified protocols.

Despite these limitations, the present study highlights the significance of cut-off thresholds. The choice of an optimal cut-off may not only be relevant in studies evaluating CTC counts [3, 6, 12], but even more in those assessing expression levels of CTC-related genes [7–9, 15]. In the present study, transcript levels of the previously investigated 6-gene panel [11] in residual WBC and the selection of a cut-off threshold led to potentially false negative results in blood samples enriched by the single-step protocol using the GEN3D6.5 microfluidic cassette. In paired samples with a more efficient depletion of WBCs—as achieved by additional DG centrifugation or by increasing the critical gap size of the separation cassette—more patient samples were assigned as *EMP2*- or *PPIC*-positive, because no gene expression in HD was observed and the calculation of a threshold was not necessary. These results may indicate smaller differential gene expression levels of *EMP2* and *PPIC* in CTCs, as—for example—of *CK19* or *SCGB2A2*. However, considering the heterogeneous biological and physical characteristics of CTCs, a reasonable compromise has to be found between WBC depletion and recovery of CTCs. Novel normalization strategies taking the individual WBC background into account [13] may be worth further investigation in future studies.

To summarize, the Parsortix® technology using the GEN3D6.5 separation cassette represents a valuable tool for subsequent gene expression analyses of multiple markers. Nevertheless, appropriate gene expression markers, assays and of cut-off threshold values need to be used and validated to enable correlation with clinical outcome in specified patient populations.

## Data Availability

The datasets generated during the current study are available from the corresponding author on reasonable request.
